# Efficacy and safety of belimumab/low-dose cyclophosphamide therapy in moderate-to-severe systemic lupus erythematosus

**DOI:** 10.3389/fimmu.2022.911730

**Published:** 2022-08-01

**Authors:** Hao Cheng, Xiao-ying Zhang, Hui-dan Yang, Zhen Yu, Cheng-lan Yan, Chong Gao, Hong-yan Wen

**Affiliations:** ^1^ Department of Rheumatology, The Second Hospital of Shanxi Medical University, Shanxi Medical University, Taiyuan, China; ^2^ Department of Pathology, Brigham and Women’s Hospital, Harvard Medical School, Boston, MA, United States

**Keywords:** belimumab, low cyclophosphamide (CYC), systemic lupus erythematosus (SLE), B cells, T cells, IL-6

## Abstract

**Objectives:**

We have reported previously that Belimumab, a human monoclonal antibody that inhibits B-cell activating factor(BAFF) could be an effective and safe option to treat Neuropsychiatric manifestations of SLE (NPSLE). To avoid inadequate efficacy of Belimumab and significant adverse events of often-used dose of cyclophosphamide (CYC) for SLE, we evaluated the efficacy, safety, and possible immune mechanisms of Belimumab treatment in combination with intermittent low-dose intravenous CYC for moderate-to-severe SLE.

**Methods:**

In this non blinded and parallel-group trial, we collected 82 cases of moderate-to-severe SLE patients, 40 received Belimumab treatment and 42 received conventional treatments as historical controls for 24 weeks. The demographic features, clinical manifestations, and laboratory indicators including peripheral blood lymphocyte subgroups or subsets were compared before and after the treatments.

**Results:**

Compared with the baseline, 6 months post Belimumab group treatment, disease activity score SLEDAI (13.78 to 3.82, P<0.05) and BILAG scores (16.40 to 5.48, P<0.05) were reduced; C3 (0.19 to 1.14, P<0.05) and C4 (0.04 to 0.22, P<0.05) increased; the absolute numbers of B and T cells were the first decreased and then significantly increased, tended to balance. Moreover, Belimumab group treatment significantly reduced the serum levels of IL-6, the ratio of B and T cells, and the proportion of infections and menstrual disorders.

**Conclusion:**

Compared with conventional treatment, Belimumab with low-dose intravenous CYC significantly reduced disease activity scores and maintained the B/T cell balance for SLE patients at 24 weeks. It was more efficacy and safe (adverse events such as infection were significantly lower). It should be the mechanism that Belimumab combined with low-dose intravenous CYC therapy restores the balance of T and B cells, which proposes a potential treatment strategyfor SLE.

## Introduction

Systemic lupus erythematosus (SLE) is a complex chronic autoimmune disease with highly variable disease manifestations and organ involvement (1–3). At present, corticosteroids and conventional immunosuppressive agents are the main treatment methods. However, they are not always effective and tolerable and they may cause the adverse events in some patients. So, active SLE with infection remains an important cause of relapse and mortality (4).

Current treatments for moderate-to-severe SLE patients include cytotoxic immunosuppressive agents, including usually middle (modified NIH regimen) and high-dose (NIH regimen) cyclophosphamide (CYC), and high-dose steroids. Remission occurs following treatment in 70-90% of patients. However, disease relapse, persistent disease activity, and treatment-associated toxicity contribute to mortality and chronic incapacity. Most deaths are associated with the treatment side effects (4, 5). Emerging biological agents have also been used for treatment of SLE. Rituximab is a chimeric monoclonal antibody targeted against CD20 which is a surface antigen present on B cells. As Rituximab (RTX) has been shown to increase BAFF levels following B cell depletion, repeated RTX treatments may result in more severe flares driven by BAFF (6). Thus, high BAFF levels post RTX could limit its effectiveness in some patients with SLE (7). Furthermore, a large, phase III, and randomized placebo-controlled trial failed to meet their primary endpoints (4, 8). Therefore, treatments with less toxic and more effective are urgently needed.

Since clinical response are extremely variable in SLE patients, no single mediator or pathway can account for the complex pathogenesis. Some studies have showed that immune dysregulation at the level of T cells, B cells, macrophages, and cytokines are closely related to SLE pathogenesis (9, 10). B cells are implicated in SLE pathogenesis by production of autoantibody, presentation of autoantigen to T cells, T cell activation, and cytokine production. Meanwhile, T cell also are critically in SLE, infiltrating widely into target organs to cause inflammation and organ damage. On the other hand, many studies of SLE have shown that T cells have many abnormalities of cytokine production and cell signaling transduction, which not only determines the abnormal differentiation of T cells, but also the overactivation of B cells (11–14).

The treatment regimen of CYC and glucocorticoids widely acted on B and T cells and significantly increased long-term efficacy (nearly 10-year period), leading to less mortality in SLE (4, 9, 15, 16). However, infection is one of the leading causes of morbidity and mortality in SLE, which has intrinsically increased risks that are expanded by immunosuppressive therapy. Belimumab, a human monoclonal antibody that inhibits B-cell activating factor (BAFF), reduces the number of circulating B cells (8–10). Although Belimumab has demonstrated efficacy both in clinical trials and in real-world settings and has a safe long-term side-effect profile, it is not a panacea for all SLE patients. In clinical trials, at least 40% of SLE patients did not demonstrate a clinically meaningful response to Belimumab that only targets BAFF (10). Also, we reported the effect of Belimumab on five patients who were unresponsive to conventional therapy. Our case reports suggest that Belimumab could be an effective and safe option to treat NPSLE, even in refractory cases, allowing to spare glucocorticoids and immunosuppressants (15). A combination therapy targeting multi-pathways and/or cells could be more effective. Therefore, we developed a novel therapy: Belimumab with a low-dose intravenous CYC for moderate-to-severe SLE.

This study is to investigate whether Belimumab with low-dose intravenous CYC is more effective and safe for SLE patients and effects of T cells and cytokines besides B cells in the peripheral blood of moderate-to-severe SLE patients. To our knowledge, we described, for the first time, the changes in disease activity, absolute numbers of peripheral lymphocyte subgroups, and serum cytokines after Belimumab (10mg/kg, the first three doses were administered once every 2 weeks and then once every 4 weeks total 8) and low-dose intravenous CYC (the first doses was 400mg and then a fixed dose of 200mg/3week) treatment in patients with SLE over a 24 weeks period.

## Methods

### Study design and participants

To assess the efficacy and safety of Belimumab combined with low-dose intravenous CYC in patients with moderate-to-severe SLE, we did a retrospective study at the Department of Rheumatology, Second Hospital of Shanxi Medical University (Taiyuan, China), with approval from the Second Hospital of Shanxi Medical University Ethics Committee (ethics number: 2019YX140). This trial was registered at the Chinese Clinical Trial Registry (ChiCTR2200055471).

We collected 40 cases of moderate-to-severe SLE patients in the Second Hospital of Shanxi Medical University from January 2021 to December 2021 into the study, who received Belimumab treatment. A total of 42 moderate-to-severe SLE patients who met the same criteria from April 2014 through June 2015 and received conventional treatments as historical controls.

All patients eligible participants were age ≥18 years and had to fulfill ≥4 of the 11 American College of Rheumatology 1997 classification criteria for SLE (17, 18), and were required to have serum positivity for antinuclear antibodies (ANAs) and/or anti-double-stranded DNA (anti-dsDNA) antibodies at the time of screening (17). All had active severe disease: defined as Systemic Lupus Erythematosus Disease Activity Index 2000 (SLEDAI-2K) score of ≥6, and either ≥1 organ system (include renal or central nervous system) with a British Isles Lupus Assessment (BILAG) A score (severe disease activity) or ≥2 organ systems with a BILAG B score (moderate disease activity). At least 1 of the BILAG A grades or ≥2 of the BILAG B grades must have been in the mucocutaneous, musculoskeletal or cardiorespiratory BILAG-2004 index domains (19–21), and coexists with another autoimmune diseases were excluded. Any known intolerance or contraindications to CYC and others DMARDs, acute infection, cancer or other malignant disease, or other connective tissue diseases were excluded from the study.

### Procedures

**Figure 1:**
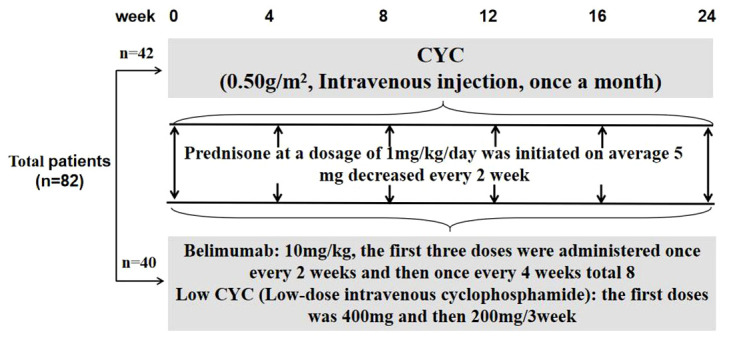
Flow chart of the study design and patient disposition.

The patients continued to receive corticosteroid and any of concomitant immunosuppressants at the same dose used before study entry. For those with high disease activity at screening, a dose increase in corticosteroid was allowed at the investigator’s discretion. Belimumab at 10 mg/kg was given intravenously at 2-week intervals for the first 3 doses and combined with low-dose intravenous CYC at the first doses was 400mg and then 3-week intervals 200mg until week 24. The CYC group included patients who received induction with monthly intravenous CYC (0.5–1.0 g/m^2^) (**Figure 1**).

The clinical follow-up assessment mainly included laboratory assessment (including serum cytokine), disease activity, occurrence of infection, and any other adverse reactions at baseline, 4, 8, 12, 24 weeks after treatment. Data for each patient was assessed by the same investigator throughout the study using the following outcome measures.

### Assessment of disease activity

The BILAG index was used to assess response, and scores were converted to numeric values (A = 9, B = 3, C = 1, D = 0, E = 0) to enable evaluation of fluctuating global summary scores (20, 21).

### Laboratory assessment

The absolute numbers of lymphocyte subpopulations in peripheral blood of these individuals were determined by flow cytometry combined with a known number of fluorescent beads (21). Anti-double-stranded DNA (anti-dsDNA) antibody titers were measured by enzyme-linked immunosorbent assay, and C3 and C4 complement levels and total serum immunoglobulin titers and Erythrocyte Sedimentation Rate (ESR) were measured by nephelometry (20, 22–24).

### Measurement of serum cytokine

The levels of serum cytokines [IL-6, IL-10, IL-17, and tumor necrosis factor (TNF)] in SLE (serums were kept at −80°C until analysis) were detected by magnetic bead-based multiplex immunoassay using Human Th1/Th2/Th17 subgroup test kit. The Bio-Plex 200 reader was used to acquire the data of cytokines, which was output as Median Fluorescence Intensity (MFI) and concentration (pg/mL) using the BioPlex Manager software (25, 26).

### Safety assessments

Liver dysfunction was defined as more than twice the upper limit of normal (AST, ALT ≤ 40 U/L). Renal dysfunction was defined as an increase in serum creatinine concentration of more than 30% above baseline levels at any study time point. Blood system toxicity refers to bone marrow suppression, such as leukopenia. Gastrointestinal reactions were nausea, vomiting, abdominal pain and other discomfort, and menstrual disorders. Serious adverse events (SAEs), infusion-related AEs (an AE occurring during or within 24 hours following completion of the infusion of a study drug), and infection-related AEs were summarized independently. Infection refers to a fever occurring, and evidence of a pathogenic infection is found (16, 27–29).

### Statistical analysis

Data were statistically significant at a value of P<0.05. Statistical analyses were performed by SPSS version 23.0 (IBM Corp, Armonk, NY, USA) and GraphPad Prism version 8.01. Normal distributed variables’ descriptive data were presented as mean and standard deviation (SD) and non-normal distributed variables were presented as median with range. Categorical variables were reported as numbers. Paired-samples t-test or paired-samples Wilcoxon test was used for comparison of changes before and after treatment. Independent-samples t-test or Mann-Whitney U test was used to compare the differences between two groups. Spearman’s rank correlation test was used to evaluate the correlation. The correlation coefficient of 0.1 to 0.3 was weak correlation, 0.3 to 0.5 was moderate correlation, and 0.5 to 1.0 was strong correlation.

## Results

Clinical and laboratory characteristics of 82 SLE patients were collected and analyzed. Among them, 42 were received formal dose of CYC treatment (CYC group), and 40 were received Belimumab with low-dose intravenous CYC (Belimumab group). The demographic and disease characteristics of the patients are presented in **Table 1**. The mean age of the patients was 30.79 ± 11.68 years. Between the two groups, the differences were not statistically significant in the general conditions and disease indicators, including SLEDAI-2K and BILAG scores, and C3, C4, as well as laboratory tests (P>0.05). The two groups were comparable.

**Table 1:** Baseline characteristics of 82 patients with SLE.

Variables	Belimumab	CYC	*P *(value)
	(N=40)	(N=42)
Age, mean ± SD (years)	30.57 ± 11.68	31.02 ± 12.04	0.619
Female, n (%)	39(97.5)	40(95.2)	0.341
History of smoking, n (%)	1(2.50)	1(2.38)	0.862
Disease duration, mean ± SD (years)	2.6 ± 2.36	2.7 ± 2.86	0.563
SLEDAI, mean ± SD	13.78 ± 2.21	12.63 ± 1.96	0.813
BILAG, mean ± SD	16.4 ± 6.52	16.0 ± 6.84	0.613
C3, mean ± SD	0.19 ± 0.11	0.21 ± 0.10	0.374
C4, mean ± SD	0.04 ± 0.09	0.05 ± 0.11	0.416
ESR, mean ± SD (mm/h)	61.81 ± 12.68	58.15 ± 11.00	0.599
CRP, mean ± SD (ng/ml)	32.57 ± 7.84	34.21 ± 10.11	0.674
ANA, n (%)	40(100)	42(100)	1.000
Anti-ds-DNA, n (%)	40(100)	42(100)	1.000
Anti-Sm, n (%)	28(70.0)	34(81.0)	0.085
Background prednisone, n (%)			
Daily prednisone use	40(100)	42(100)	0.875
>7.5 mg/d at baseline	40(100)	42(100)	0.875
Prednisone, mean ± SD (mg/d)	41.4 ± 16.2	42.7 ± 15.6	0.762
Background immunosuppressive drug, n (%)			
CYC, n (%)	40(100)	42(100)	0.875
MMF, n (%)	36(90.0)	37(88.1)	0.657
HCQ, n (%)	38(95.0)	42(100)	0.375
LEF, n (%)	2(5.0)	4(9.5)	0.215
AZA, n (%)	1(2.5)	0(0)	0.425

Except stated otherwise, values are reported as mean ± SD or number (%). ANA, Antinuclear antibodies; Anti-ds-DNA, anti-double-stranded DNA; CYC, cyclophosphamide; MMF, Mycophenolate mofetil; HCQ, Hydroxychloroquine; LEF, leflunomide; AZA, azathioprine.

### Equal effects of belimumab/low-dose CYC and conventional CYC therapy

**Figure 2:**
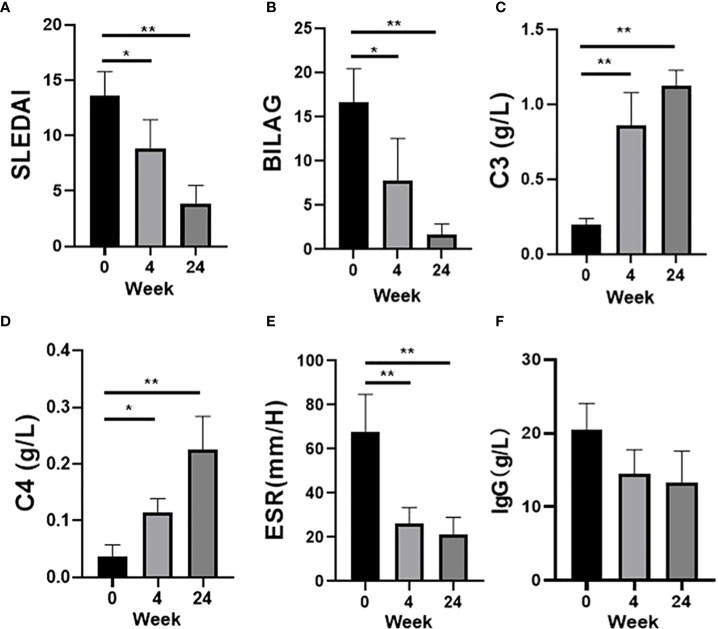
Effects of Belimumab after 6 months as compared to the baseline occurring before treatment. **(A, B)** Belimumab significantly improved SLEDAI and BILAG score. **(C, D)** C3 and C4 showed a significantly increased. **(E, F)** ESR and IgG show a significantly downward (*p<0.05, **p<0.01).

First, we analyzed the efficacy of Belimumab co-treated with low-dose intravenous CYC for 6 months. Compared with the baseline, Belimumab group reduced significantly SLEDAI-2K and BILAG scores (**Figure 2B**) of SLE patients, and low complement (C3 and/or C4, < lower limit of normal) or high ESR demonstrated an improvement (**Figure 2E**). Similarly, immunoglobulin IgG decreases, but the difference was not statistically significant (**Figure 2**).

**Figure 3:**
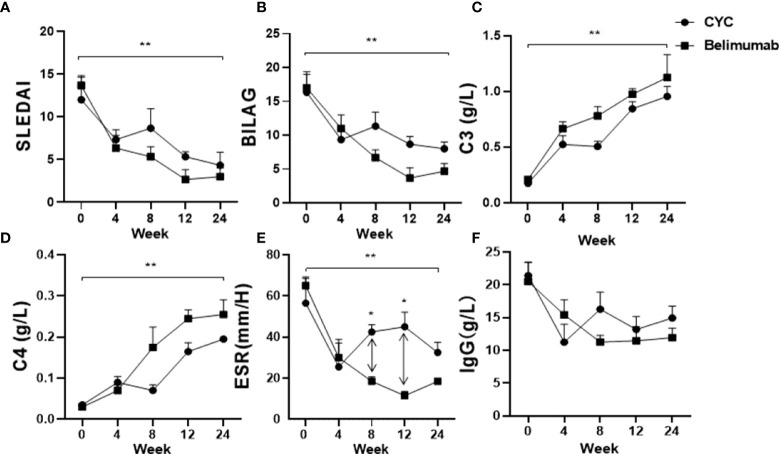
Effects of Belimumab and CTX treatment after 6 months as compared to the baseline occurring before treatment. **(A, B)** Two groups significantly improved SLEDAI and BILAG score. **(C, D)** C3 and C4 of both groups showed a significantly increased. **(E, F)** ESR and IgG show a significantly downward (*p<0.05, **p<0.01). Belimumab Group: Belimumab combined with low-dose CTX; CTX Group: Standard-dose of CTX.

At the same time, we used conventional therapy alone (CYC group) as controls, and compared the efficacy Belimumab group. Compared with the baseline, after 24 weeks of treatment, both two treatments reduced the SLEDAI-2K and BILAG scores significantly (**Figure 3B**), but the difference between the two groups was no statistical significance. There were significant improvements in C3, C4 after both treatments (P <0.05), but no statistically significant differences between the two groups (**Figure 3D**). However, ESR in the CYC group was significantly higher than that in Belimumab group at 8 and 12 weeks. And there was no statistical difference in other follow-up nodes between the two groups (**Figure 3**). Immunoglobulin IgG was no significant difference between the 2 groups of patients (**Figure 3**). All above suggest that Belimumab with low-dose CYC can achieve the equal effects of conventional CYC therapy.

### Changes of peripheral blood lymphocyte levels

**Figure 4:**
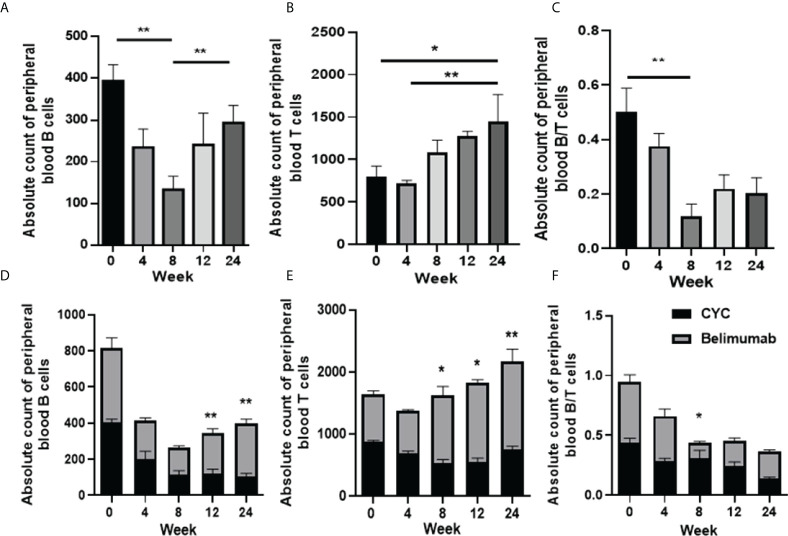
Absolute numbers of B and T cell in SLE patients before and after therapy was analyzed by flow cytometry (FCM). **(A–C)** The absolute number of B, T cells and the B/T ratio of the Belimumab group. **(D-F)** The absolute number of B, T cells and the B/T ratio of two treatment regimens. *p<0.05 and ** p<0.01 are significantly compared to the baseline. Belimumab Group: Belimumab combined with low-dose CTX; CTX Group: Standard-dose of CTX.

First, we analyzed the changes of peripheral blood B cells, T cells and their ratios before and after the treatment of the Belimumab group. We found that the absolute number of B cells decreased from the baseline at 4-8 weeks of the treatment, and then gradually increased to 12 weeks after the treatment, with significant differences (**Figure 4A–F**). At the same time, T cells decreased slightly after 4 weeks of treatment, with no statistical difference from baseline. Subsequent treatment up to 24 weeks led to a gradual increase in T cells, and a statistical difference from baseline and 4 weeks (**Figure 4**). The B/T ratio tended to balance at 24 weeks, but compared with the baseline, there were statistical differences when compared with the B/T ratio at 8 week (**Figure 4**). Second, we analyzed and compared to the effect of two treatment regimens on B/T cell balance. Our research suggested that, the absolute numbers of B and T cells of CYC group of patients decreased significantly as compared to those before with treatment, there was a continuous decrease, especially in T cells. However, the Belimumab group showed a slight decrease in B and T cells in the early stage of treatment (4-8 weeks) and gradually increased as treatment continued. After 8 weeks, B and T cells were statistically different between the two groups (**Figure 4E**). The ratio of the two is maintained in a relative equilibrium state. The imbalance of B and T cells was obvious at 8 weeks after CYC group treatment (**Figure 4**).

### Changes of cytokines Levels

**Figure 5:**
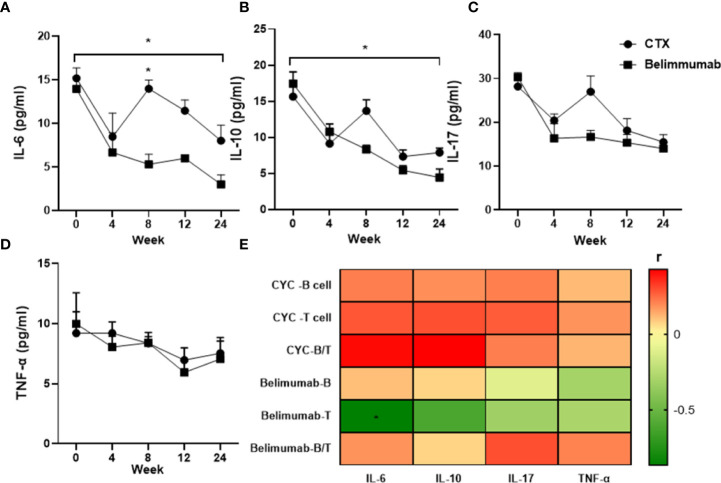
Decrease in levels of serum pro-inflammatory cytokines of SLE patients in both groups after 6-months treatment. Mean concentrations (pg/mL) of serum cytokines were quantified by ELISA: **(A–D)** IL-6, IL-10, IL-17, TNF. **(E)** Heatmap showed that the serum levels of IL-6, IL-10, IL-17, TNF have significant correlation with the peripheral blood lymphocyte (B cell, T cell) and B/T (*r>0.5 or<-0.5). Spearman’s correlation test was used. T: Total CD3+T cells, B: Total B cells. B/T the radio of B cell versus T cell. *p<0.05 is statistically significant compared to the baseline. Belimumab Group: Belimumab combined with low-dose CTX; CTX Group: Standard-dose of CTX.

In this study, after Belimumab treatment for 24 weeks, the serum levels of IL-6 and IL-10 were significantly decreased comparing with the baseline. Serum levels of IL-6 of CYC group showed the most rapid and prominent reduction, but increased again at week 8 of treatment and was significantly different from Belimumab. Similarly, Serum levels of IL-10 increased again after 8 weeks of treatment, but there was no statistical difference between the two groups. After 24 weeks of treatment, IL-6 and IL-10 were significantly decrease than baseline in both groups, with significant differences (**Figures 5A, B**). However, serum levels of IL-17 and TNF showed no difference after treatment (**Figures 5C, D**). We analyzed the correlation of the serum levels of these cytokines with B cells, T cells and their ratios in the groups. And we found that the serum levels of IL-6 were significantly correlated T cells in Belimumab group. It is suggested that our therapeutic regimen may affect the absolute T cells through IL-6. But other indicators were not significant correlation (**Figure 5E**).

### Safety

The safety of Belimumab has been demonstrated in the treatment of SLE (25, 26). In this study, it had no serious adverse events. However, the incidence of adverse events of infection and menstrual disorders were significantly increased in the CYC group, which was significantly higher than that in Belimumab group. In the CYC group, 2 patients did not complete follow-up treatment due to severe infection; 10 patients were re-hospitalized due to infection; the remaining infected cases were cured successfully by antibiotic treatment in outpatient. Menstrual disorders, gastrointestinal reactions, leukopenia, and liver damage were improved after treatment by a specialist (**Table 2**).

**Table 2:** Adverse events occurred in both groups.

Variables	Belimumab	CYC	*P (value)*
	(N=40)	(N=42)
Death	0(0)	0(0)	1.000
Increased serum creatinine	0(0)	0(0)	1.000
Abnormal liver function tests	2(5.0)	13(31.0)	0.0903
Infection	4(10.00)	27(67.5)	<0.001
serious infection	0(0)	2(4.7)	0.778
menoxenia	3(7.5)	21(50.0)	<0.05
leukopenia	0(0)	2(4.7)	0.778
Gastrointestinal reaction	6(15.0)	16(38.1)	0.0681
hemorrhagic cystitis	0(0)	0(0)	1.000

## Discussion

This is the first retrospective study to analyze the efficacy and safety of standard-dose CYC versus Belimumab combined with low-dose CYC in the treatment of moderate to severe SLE. Although the study was not powerful enough to fully assess efficacy and was primarily designed to assess safety, the overall efficacy was consistent with those in many published literatures, and our therapy was no significant different from conventional treatment (4, 10, 16). The Belimumab with low-dose CYC significantly reduced the risk of infection and menstrual disorders, as well as hematological toxicity and gastrointestinal reactions compared with the standard dose. In addition, decrease in IL-6 may be a key step in keeping B/T cell homeostasis.

The pathogenesis of SLE involves abnormalities of several components of the immune system, including B cells, T cells, cytokines and growth factors (11, 14). Intermittent intravenous impulse therapy with high-dose CYC combined with glucocorticoid has been the classic treatment for moderate and severe SLE (16, 30). While SLE patients applied CYC to induce often require high-dose and long-term treatment for disease remission. However, this treatment often leads to many adverse reactions, including leukopenia, infection, reproductive toxicity, hair loss and gastrointestinal reactions (11). In recent years, a better understanding of the pathogenesis of SLE has led to the introduction of several biologics, such as rituximab (RTX) and Belimumab, which are available in clinical practice (4, 9, 31). Belimumab is the only targeted biologic approved for the treatment of lupus. Based on the results of randomized clinical trials (RCTs) and real-life experience, Belimumab is particularly effective in patients with active, serologically active disease, and early use leads to a better clinical response (10, 32). But the addition of Belimumab to CYC, rituximab, and glucocorticoid treatment regimens targeting different mechanisms of action of B cells did not achieve better efficacy and resulted in an increase in serious infectious adverse events (7, 33).

In recent years, many clinical studies have shown that low-dose CYC combined with intermittent intravenous impulse therapy of glucocorticoid is equivalent to that of high-dose regimen in the treatment of low-moderate SLE, with fewer adverse reactions (16). According to the lymphocyte cell cycle, our research group gave a low dose of CYC 200mg every 3 weeks, but the disease might be poorly controlled for moderate- severe SLE (34). Therefore, our research protocol applied a low dose of CYC combined with Belimumab for the first time.

In this study, we retrospectively analyzed that both treatment regimens were effective, especially in reducing disease activity scores and lupus indicators. We found that there was no difference between the two groups. Similarly, we found that both treatment regiments affected T cells besides B cells. Importantly, B cell function requires a combination of antigen presentation, cytokine production, and T cell activation and polarization. Studies have shown that B cell depletion therapy has a significant effect on diseases, which was previously thought to be primarily driven by T cells (35, 36). T lymphocytes play a fundamental role and are believed to trigger SLE disease, especially enhancing autoantibody production by B cells. Dysregulation of transcription factors and cytokines in B cells can lead to abnormal maturation of B cells and the production of autoantibodies (36). Targeted blocking of B-cell-related cytokines have an obvious effect on down-regulating the strong inflammation immune response (17, 26). During the treatment of Belimumab combined with low-dose CYC, B and T cells decreased slightly from baseline after 4-8 weeks of treatment, but increased from baseline after 24 weeks of treatment, and the ratio of the two cells tended to balance. It is suggested that the treatment scheme acts on both B cells and T cells, and their equilibrium and interaction mechanism may be affected by some key cytokines.

IL-6 levels are found to be raised in the serum of SLE patients and it has both inflammatory and anti-inflammatory effects (17). IL-6, IL-10, TNF, IL-17, and immunoglobulin levels were reduced by our therapy. Interestingly, serum levels of IL-6 and IL-10 showed the most rapid and significantly earlier decline and serum levels of TNF and IL-17 also had moderate and slower reduction. A decrease in IL-6 levels most likely reflects a general reduction in inflammatory activity and in clinical markers of disease activity, which is known to play an important role in T-helper cell differentiation, as shown here and in previous observational studies (37, 38). And there is evidence that IL-6 can induce and magnify the production of autoantibodies in autoimmune diseases (37). So, it is important that an early decrease in IL-6 levels within 6 months of Belimumab/low-dose CYC treatment is associated with sustained remission of SLEDAI, BILAG scores, and clinical indicators. The consistency of these associations supports the role of IL-6 as a useful marker of inflammation in SLE patients, and early decline may thus indicate suitability for continued therapy. Tocilizumab, which also targets the IL-6 receptor, is already used to treat severe active RA, systemic and polyarthritis JIA, and giant cell arteritis (GCA) (39–41). Tocilizumab has been shown to be effective in the treatment of juvenile systemic lupus erythematosus (JSLE), especially in patients with SLE involving the central nervous system (15, 42). In the study of Shirota et al. Tocilizumab was used in 15 SLE patients with mild-moderate disease activity with a reduction in the activity of T and B cells (43). A phase II randomized trial of CNTO 136 in patients with active lupus nephritis is completed and the results are awaited. Successful cases of tozizumab and tacrolimus in the treatment of patients with rheumatoid arthritis complicated by SLE have also been reported (44). Belimumab combined with IL-6 inhibitor of IL-6 receptor may be a novel treatment for SLE.

Despite the retrospective nature, our results indicate that both treatments can induce remission of the disease. Two patients in the standard-dose CYC group stopped follow-up treatment due to severe infection. No patients in the Belimumab combined with low-dose CYC group were interrupted by the drug side effects of this treatment. Most of the adverse events in patients who received were low grade. Importantly, it demonstrated multidimensional effects on patients with SLE. Therefore, Belimumab combined with low-dose CYC is safe, feasible and may become the optimal regimen for the treatment of SLE, especially for the moderate-to-severe SLE.

There are limitations in our study. This was small sample size study that enrolled a limited number of patients with SLE. More clinical and laboratory studies using a large size samples and long-term observation are needed to confirm importance of the B/T balance in SLE. From a clinical point of view, treatment is controlled by organ involvement and predictors in different disease subgroups need to be identified to facilitate an individualized management approach. We were unable to analyze concomitant and prior medications other than glucocorticoids. Observing design can also be considered a disadvantage, for example, decisions directed by the treating physician rather than the purpose of the study may hinder standardization of background treatment. However, patient cases represent real life scenarios and follow-up represents current clinical practice, both of which can also be considered as strengths of the study. Furthermore, we only retrospective selected patients with conventional immunosuppressants as comparators, specific csDMARDs combined with Belimumab should be optimized in the future. This was a pilot study designed based on short-term efficacy and safety. To provide preliminary data that could drive more conclusive testing. Therefore, high-quality, large-scale, multicenter randomized controlled trials with longer follow-up are needed to further compare safety and efficacy.

## Conclusions

Our study shows at first time that Belimumab combined with low-dose CYC treatment can improve disease activity and clinical performance without severe adverse events. In particular, the adverse events of infection were far less than those of conventional treatment. In addition to targeting B cells, Belimumab also appears to affect T cells and their subsets *via* IL-6, acting as an immune-balancing therapy. However, further study should be done using large-size samples and a well-designed, double-blind, randomized controlled trial is needed.

## Data availability statement

The original contributions presented in the study are included in the article/**Supplementary Material**. Further inquiries can be directed to the corresponding author.

## Ethics statement

The studies involving human participants were reviewed and approved by the Second Hospital of Shanxi Medical University Ethics Committee (ethics number: 2019YX140). The patients/participants provided their written informed consent to participate in this study.

## Author contributions

HC and X-YZ performed the data analyses and wrote the manuscript. H-DY, ZY, and C-LY participated in the collection of samples and clinical data. CG participated in the study design and revising of the manuscript. H-YW provided intellectual input and supervision throughout the study and made a substantial contribution to manuscript drafting. All authors contributed to the article and approved the submitted version.

## Funding

This study was supported from Key Scientific Research Project of Medical Science of Shanxi Province (2021XM08), Basic Research Youth Project of Shanxi Province (202103021223442) and 2020 Shanxi Province Emerging Industry Leadership Project (2020-15).

## Conflict of interest

The authors declare that the research was conducted in the absence of any commercial or financial relationships that could be construed as a potential conflict of interest.

## Publisher’s note

All claims expressed in this article are solely those of the authors and do not necessarily represent those of their affiliated organizations, or those of the publisher, the editors and the reviewers. Any product that may be evaluated in this article, or claim that may be made by its manufacturer, is not guaranteed or endorsed by the publisher.
