# Supplementary material for: Efficacy and safety of belimumab/low-dose cyclophosphamide therapy in moderate-to-severe systemic lupus erythematosus
Source: Front Immunol. 2022 Aug 1;13:911730. doi: 10.3389/fimmu.2022.911730 (PMC9376229; doi:10.3389/fimmu.2022.911730)
Supplement: Supplementary file 1 [file DataSheet_1.docx]

**Changes in prednisones dose**

At baseline, all moderate-to-severe SLE patients (40patients in the belimumab group and 42 in the CYC group) receiving prednisones at a dosage of >7.5 mg/day (Table 1). More patients who received belimumab were able to reduce their prednisones dosage by ≥50% at week 24 as compared with CYC group (24.2% versus 21.9%, P＞0.05), but there were no statistical differences between the two groups. There was a difference of 550.30 mg in the mean ± SD cumulative dose of prednisones at 24 weeks, the same was that this difference did not statistical significance (2985.5± 1554.32 for belimumab and 3535.8 ± 1,736.83 for CYC group; P＞0.05).
